# Acute kidney injury (AKI) in patients with Covid-19 infection is associated with ventilatory management with elevated positive end-expiratory pressure (PEEP)

**DOI:** 10.1007/s40620-021-01100-3

**Published:** 2021-06-25

**Authors:** Davide Ottolina, Luca Zazzeron, Letizia Trevisi, Andrea Agarossi, Riccardo Colombo, Tommaso Fossali, Mattia Passeri, Beatrice Borghi, Elisabetta Ballone, Roberto Rech, Antonio Castelli, Emanuele Catena, Manuela Nebuloni, Maurizio Gallieni

**Affiliations:** 1 Department of Anesthesia and Critical Care, “Luigi Sacco” Hospital, ASST Fatebenefratelli-Sacco, Via G.B. Grassi, 74, 20157 Milan, Italy; 2 Department of Anesthesia, Critical Care, and Pain Medicine, Massachusetts General Hospital, Boston, MA USA; 3 Department of Global Health and Social Medicine, Harvard Medical School, Boston, MA USA; 4 ‘L. Sacco’ Department of Biomedical and Clinical Sciences, Università degli Studi di Milano, Milan, Italy; 5 Pathology Unit, “L. Sacco” Hospital, ASST Fatebenefratelli-Sacco, Milan, Italy; 6 Nephrology and Dialysis Unit, “L. Sacco” Hospital, ASST Fatebenefratelli-Sacco, Milan, Italy

**Keywords:** Covid-19, ARDS, AKI, PEEP, Intensive care

## Abstract

**Background:**

Acute kidney injury (AKI) in Covid-19 patients admitted to the intensive care unit (ICU) is common, and its severity may be associated with unfavorable outcomes. Severe Covid-19 fulfills the diagnostic criteria for acute respiratory distress syndrome (ARDS); however, it is unclear whether there is any relationship between ventilatory management and AKI development in Covid-19 ICU patients.

**Purpose:**

To describe the clinical course and outcomes of Covid-19 ICU patients, focusing on ventilatory management and factors associated with AKI development.

**Methods:**

Single-center, retrospective observational study, which assessed AKI incidence in Covid-19 ICU patients divided by positive end expiratory pressure (PEEP) tertiles, with median levels of 9.6 (low), 12.0 (medium), and 14.7 cmH_2_O (high-PEEP).

**Results:**

Overall mortality was 51.5%. AKI (KDIGO stage 2 or 3) occurred in 38% of 101 patients. Among the AKI patients, 19 (53%) required continuous renal replacement therapy (CRRT). In AKI patients, mortality was significantly higher versus non-AKI (81% vs. 33%, p < 0.0001). The incidence of AKI in low-, medium-, or high-PEEP patients were 16%, 38%, and 59%, respectively (p = 0.002). In a multivariate analysis, high-PEEP patients showed a higher risk of developing AKI than low-PEEP patients (OR = 4.96 [1.1–21.9] 95% CI p < 0.05). ICU mortality rate was higher in high-PEEP patients, compared to medium-PEEP or low-PEEP patients (69% vs. 44% and 42%, respectively; p = 0.057).

**Conclusion:**

The use of high PEEP in Covid-19 ICU patients is associated with a fivefold higher risk of AKI, leading to higher mortality. The cause and effect relationship needs further analysis.

**Graphic abstract:**

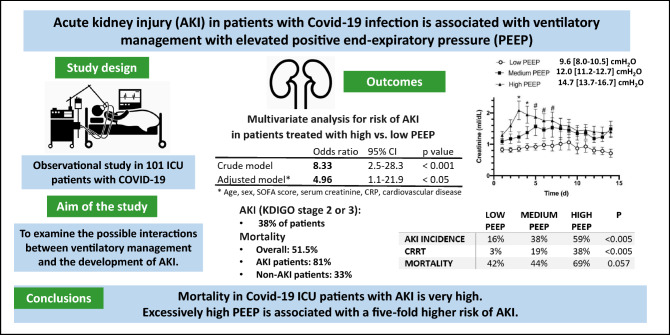

**Supplementary Information:**

The online version contains supplementary material available at 10.1007/s40620-021-01100-3.

## Introduction

The severity of the disease caused by the novel ‘severe acute respiratory distress syndrome coronavirus 2’ (SARS-CoV-2) varies largely from asymptomatic cases to more severe presentations. Five to ten percent of hospitalized patients require admission to the intensive care unit (ICU) [1, 2], due to acute hypoxemic respiratory failure requiring mechanical ventilation.

Based on pre-existing evidence and guidelines on acute respiratory distress syndrome (ARDS), mechanical ventilation of Covid-19 patients with ARDS involves the use of low tidal volume (Vt), positive end-expiratory pressure (PEEP), low driving pressure and low plateau pressure [3]. However, various authors reported that Covid-19 ARDS often presents with preserved lung mechanics and well-aerated lungs on computed tomography (CT) scan [4]. To explain the severe hypoxemia conflicting with the respiratory system mechanics and CT findings, alternative mechanisms such as impairment of hypoxic pulmonary vasoconstriction and micro-thrombi formation in the pulmonary circulation have been proposed [5]. Based on these observations, specific ventilatory management, including low PEEP (8–10 cmH_2_O) and more liberal Vt (7–8 mL/kg), has been proposed [5, 6].

Acute kidney injury (AKI) has been reported in 20–30% of Covid-19 ICU patients [7, 8]. Multiple mechanisms have been proposed, including cytokine storm [9], direct virus-mediated renal damage [10], and pre-renal etiology due to aggressive diuretic use [11]. Previous studies found no clear correlation between ventilatory management and the risk of developing AKI in patients with ARDS [12]. Whether there is any relationship between ventilatory management and AKI development in Covid-19 ICU patients has not been established yet.

In this single-center retrospective study, we describe the clinical course and outcome of 101 Covid-19 ICU patients, focusing on ventilator management and factors associated with AKI development.

## Methods

### Study design and data collection

This single-center retrospective observational study included 101 consecutive adult patients admitted to the Sacco Hospital ICU in Milan from February 21st to April 28th, 2020. The Ethics Committee of L. Sacco Hospital approved the study, and informed consent was waived considering the observational, non-interventional nature of the research and the ICU clinical setting (Comitato Etico interaziendale Area 1, Milan, approval number 2020/ST/116).

Demographic and clinical characteristics of all patients were collected and recorded in a dedicated database. Daily laboratory data, including basic metabolic panel, complete blood count, liver function tests, coagulation profile, and arterial blood gas analysis, were recorded for the entire duration of the ICU stay. Ventilatory settings and adjunctive therapies such as pronation, neuromuscular blockade, and inhaled nitric oxide use were also recorded. The static (C_stat_) and dynamic (C_dyn_) respiratory system compliance were calculated according to standard formulae (see Supplement details). AKI was defined according to the 2012 Kidney Disease: Improving Global Outcomes (KDIGO) clinical practice guidelines [13]. The KDIGO guidelines stage AKI according to severity (stages 1–3). In this study, we considered only patients with stage 2 (serum creatinine 2.0–2.9 times of baseline values, urine output < 0.5 mL/kg/h for ≥ 12 h) and stage 3 (serum creatinine three times of baseline values, or ≥ 4.0 mg/dL (≥ 353.6 μmol/L) increase, or the initiation of renal replacement therapy (RRT); urine output < 0.3 mL/kg/h for ≥ 24 h or anuria ≥ 12 h).

Autopsy renal samples of two patients who died with Covid-19 and with severe AKI were analyzed. Renal tissues were fixed in formalin; the histological examination was performed on PAS-stained slides. In addition, immunohistochemistry for Sars-CoV-2 (nucleocapsid protein monoclonal antibody, Novus Biological) was performed according to the Ventana-Roche protocol.

### Statistical analysis

All descriptive data are expressed as median [25th–75th inter quartile range (IQR)] unless specified otherwise. Categorical data were compared via χ^2^ test or Fisher’s exact test, as appropriate. Between-group differences of continuous variables were analyzed with Mann–Whitney U test or non-parametric one-way ANOVA, as appropriate. A two-way ANOVA was used for between-group comparison over time. We used logistic regression models to assess the association between AKI within 7 days after admission and the average PEEP level applied during the first 7 days after admission. We modeled the probability of having AKI-Injury or failure against the likelihood of AKI-Risk and no-AKI. We performed multivariate analyses, adjusting for confounders associated with both exposure (PEEP) and outcome (AKI). We compared models with different baseline characteristics using the Akaike information criterion. We derived odds ratios, 95% CIs, and p values (Supplemental Material). Statistical analysis was performed using SAS 9.2 and GraphPad 8.0. Statistical significance was defined as a p value of less than 0.05.

## Results

### Study population

One hundred and one patients were included in the study. The majority of the patients were male (77%), and the median age was 61 [53–68] years. Cardiovascular disease was the most common comorbidity, present in 51% of the study population. Sixty-one patients were transferred from outside hospitals, and 25 had already been admitted to an ICU for a median ICU stay before the transfer of 2 [0–16] days. The overall mortality rate was 51.5%. Patients who died had a higher incidence of cardiovascular disease and cancer at baseline and were more likely to be male. Inflammatory markers on admission, including C-reactive protein (CRP), lactate dehydrogenase (LDH), and D-dimer, were elevated, but there was no significant difference between survivors and non-survivors (Table 1).

**Table 1:** Demographic and clinical characteristics on admission of the study population

	Overall population (n = 101)	Survivors (n = 49)	Non-survivors (n = 52)	p
Baseline features				
Age (years)	101/101 61 [53–68]	49/49 57 [46–64]	52/52 63 [60–70]	0.096
Male sex (n-%)	101/101 78 (77)	49/49 30 (61)	52/52 48 (92)	0.0002
BMI	95/101 28 [25–31]	47/49 28 [26–32]	48/52 28 [25–31]	0.760
Smoking, n (%)	100/101 5 (5%)	48/49 2 (4)	52/52 3 (6)	0.202
Cardiovascular disease, n (%)	100/101 51 (51)	48/49 19 (40)	52/52 32 (62)	0.028
Chronic lung disease, n (%)	100/101 7 (7)	48/49 3 (6)	52/52 4 (8)	0.999
Immunodepression, n (%)	100/101 4 (4)	48/49 1 (2)	52/52 3 (6)	0.618
Diabetes, n (%)	100/101 14 (14)	48/49 7 (15)	52/52 7 (13)	0.872
Cancer, n (%)	100/101 6 (6)	48/49 0 (0)	52/52 6 (12)	0.027
SOFA score	101/101 9 [7–11]	49/49 8 [4–11]	52/52 9 [8–11]	0.346
Ventilatory and laboratory data				
Vt (mL/kg IBW)	81/101 7.6 [7.0–8.2]	37/49 7.8 [7.4–8.9]	44/52 7.4 [6.9–8.1]	0.226
PEEP (cmH_2_O)	94/101 13 [12–16]	46/49 13 [10–15]	48/52 14 [12–18]	0.101
Peak pressure (cmH_2_O)	79/101 32 [29–35]	35/49 31 [28–34]	44/52 33 [30–35]	0.024
Arterial pH	96/101 7.35 [7.29–7.42]	47/49 7.39 [7.31–7.45]	49/52 7.32 [7.27–7.37]	0.009
PaCO_2_ (mmHg)	96/101 46 [40–53]	47/49 45 [38–50]	49/52 46 [42–56]	0.360
FiO_2_ (%)	95/101 0.8 [0.6–0.9]	46/49 0.7 [0.6–0.9]	49/52 0.8 [0.7–0.9]	0.170
PaO_2_ (mmHg)	96/101 86 [72–106]	47/49 80 [67–115]	49/52 87 [74–101]	0.310
PaO_2_:FiO_2_	95/101 113 [92–152]	46/49 113 [92–176]	49/52 113 [93–142]	0.921
Lactate (mmol/L)	92/101 1.3 [1.0–1.5]	46/49 1.1 [0.9–1.4]	46/52 1.3 [1.2–1.7]	0.013
WBC (*10^9^/L)	82/101 8715 [6020–11900]	37/49 8390 [5760–11900]	45/52 8750 [6390–11890]	0.825
Neutrophils (%WBC)	75/101 88 [80–91]	34/49 86 [81–91]	41/52 90 [79–91]	0.201
Lymphocytes (%WBC)	73/101 6 [4–12]	34/49 8 [6–15]	41/52 6 [4–11]	0.016
Platelets (*10^9^/L)	82/101 227 [172–297]	37/49 232 [163–289]	45/52 200 [176–297]	0.508
D-Dimer (µg/L)	49/101 2042 [1169–5202]	27/49 1944 [1015–7000]	22/52 2069 [1169–5202]	0.486
LDH (U/L)	58/101 531 [447–664]	28/49 523 [416–643]	30/52 561 [449–691]	0.602
CRP (mg/L)	74/101 171 [88–288]	35/49 137 [67–254]	39/52 231 [109–307]	0.106
PCT within 48 h (µg/L)	83/101 0.6 [0.2–1,7]	44/49 0.3 [0.1–1.0]	39/52 0.9 [0.2–2.0]	0.142
Creatinine (mg/dL)	89/101 0.9 [0.7–1.2]	42/49 0.9 [0.7–1.1]	47/52 1.0 [0.8–1.3]	0.369
BUN (mg/dL)	48/101 50 [30–64]	24/49 48 [30–58]	24/52 50 [31–80]	0.999
Outcomes				
AKI-Risk, n (%)	14/96 (15)	8/47 (17)	6/49 (12)	
AKI-Injury, n (%)	8 (8)	1 (2)	7 (14)	
AKI-Failure, n (%)	28 (29)	6 (13)	22 (45)	
AKI-Injury + Failure, n (%)	36 (38)	7 (15)	29 (59)	< .0001
CRRT, n (%)	19 (20)	5 (11)	14 (29)	0.028
ICU LOS (days)	15 [9–21]	14 [9–25]	15 [8–20]	0.822
Mechanical ventilation (days)	90/101 13 [8–19]	38/49 11 [8–19]	52/52 14 [6–21]	0.671
ICU mortality, n (%)	52/101 (51)	0 (0)	52 (100)	NA

Data represent number of patients and percentage (%) or median [IQR]. For each parameter, if missing values are present, the number of available data is expressed. p values represent comparisons between survivors and non-survivors, Mann–Whitney U test, χ^2^ test, or Fisher’s exact test, as appropriate

*BMI* body mass index, *SOFA* sequential organ failure assessment, *Vt* tidal volume, *PEEP* positive end expiratory pressure, *PaCO*_*2*_ arterial carbon dioxide partial pressure, *PaO*_*2*_ arterial oxygen partial pressure, *FiO*_*2*_ fraction of inspired oxygen, *WBC* White blood cell count, *LDH* lactic dehydrogenase, *CRP* C-reactive protein, *PCT* procalcitonin, *AKI* acute kidney injury, *CRRT* continuous renal replacement therapy, *ICU LOS* ICU length of stay

### AKI: incidence, mortality, and histopathologic features

**Fig. 1:**
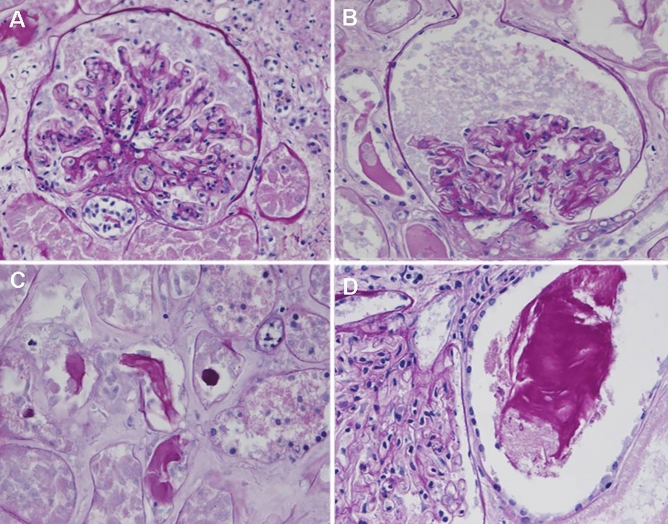
Representative post-mortem histological features in kidneys of patients with AKI-associated severe Covid-19. **A**, **B** Glomerular tuft collapse to the vascular pole, without hyperplasia or hypertrophy of podocytes. **C**, **D** Interstitial edema and tubular casts, without inflammation. Most of the tubules are lytic because of autopsy samples. Thus, tubular epithelial cells are not evaluable. **A**–**D** PAS staining, OM × 20

Among 96 patients in whom creatinine data were available, 36 (38%) developed AKI (KDIGO AKI stage 2 or 3) within 28 days of ICU stay (Table 1). Among the 36 patients who developed AKI, 19 (53%) required continuous renal replacement therapy (CRRT). Patients who developed AKI (AKI-group) had a higher SOFA score (10 [8–12] vs. 8 [5–11], p = 0.049) and higher serum creatinine at admission (1.0 [0.9–1.9] vs. 0.8 [0.7–1.1] mg/dL, p = 0.018) compared with patients who did not develop AKI (no-AKI-group). ICU mortality in AKI group was significantly higher, compared with the no-AKI group: 81% vs. 33%, p < 0.0001. No difference in the number of patients transferred from another ICU was found in the two groups (Table 2). The histopathological findings observed in the post-mortem kidneys of two Covid-19 ICU patients with AKI (Fig. 1) were glomeruli with mild or moderate tuft collapse but without hypertrophy or hyperplasia of the overlying visceral epithelium; other features were interstitial expansion by edema and tubules with protein casts. No inflammation or vascular lesions were found. Immunohistochemistry for SARS-CoV-2 antigens gave negative results.

**Table 2:** Clinical features and outcomes in patients who did or did not develop AKI

	AKI (n = 36)	No-AKI (n = 60)	p
Baseline features			
Age (years)	36/36 62 [58–69]	60/60 61 [48–68]	0.413
Male sex (n-%)	32 (89)	43 (72)	0.048
BMI	35/36 29 [26–33]	57/60 28 [25–31]	0.388
Smoke, n (%)	3(8)	2 (3)	0.304
Cardiovascular disease, n (%)	23 (64)	26 (43)	0.051
Chronic lung disease, n (%)	3 (8)	3 (5)	0.669
Immunodepression, n (%)	1 (3)	3 (5)	1.000
Diabetes, n (%)	7 (19)	5 (8)	0.125
Cancer, n (%)	2 (6)	4 (7)	0.999
SOFA score	36/36 10 [8–12]	60/60 8 [5–11]	0.049
Patients transferred from another ICU, n (%)	25 (69)	31 (52)	0.087
Laboratory data			
PaO_2_:FiO_2_	36/36 108 [78–128]	59/60 118 [93–173]	0.109
Creatinine admission (mg/dL)	33/36 1.0 [0.9–1.9]	56/60 0.8 [0.7–1.1]	0.018
BUN (mg/dL)	16/36 60 [38–83]	32/60 43 [30–56]	0.226
CRP (mg/L)	33/36 238 [129–294]	40/60 141 [68–255]	0.027
D-Dimer (µg/L)	17/36 1417 [1105–4075]	32/60 2045 [1394–10353]	0.846
Lactate (mmol/L)	32/36 1.3 [1.1–1.6]	58/60 1.2 [0.9–1.4]	0.390
WBC (*10^9^/L)	34/36 8565 [6390–12350]	48/60 9225 [5970–11895]	0.656
Neutrophils (%WBC)	32/36 89.3 [83.1–91.2]	43/60 86.7 [77.0–91.6]	0.574
Lymphocytes (%WBC)	31/36 6.0 [4.1–12.0]	42/60 6.9 [4.5–14.6]	0.122
Ventilatory and hemodynamic data			
PEEP at admission ICU (cmH_2_O)	35/36 14.0 [12.0-1m8.0]	59/60 12.5 [10.0–14.5]	0.019
Mean PEEP (cmH_2_O)	36/36 14.5 [12.4–17.0]	60/60 12.1 [10.4–14.3]	0.003
C_dyn_ (mL/cmH_2_O)	34/36 31.4 [26.4–37.9]	44/60 31.3 [26.4–35.3]	0.754
Neuromuscular blockade, n (%)	35 (97)	48 (80)	0.028
Neuromuscular blockade duration (days)	36/36 9.0 [5.5–12.0]	60/60 5.5 [1–10.5]	0.036
Prone positioning ICU, n (%)	27 (75)	33 (55)	0.050
Prone positioning duration (days)	3 [1–5]	1 [0–3]	0.015
Nitric oxide use, n (%)	3 (8)	8 (13)	0.528
Diuretic use, n (%)	34 (94)	55 (92)	0.612
Diuretic duration (days)	36/36 7 [3–13]	60/60 8 [3–13]	0.542
Cumulative urine output (L/28 days)	36/36 19 [4–33]	59/60 27 [12–43]	0.109
Fluid balance (L/28 days)	34/34 1.3[(-5.3)-(+ 4.5)]	58/60 -0.5 [(-5.3)-(+ 2.6)]	0.091
Outcomes			
ICU LOS (days)	36/36 15 [10–19]	60/60 13 [7–21]	0.208
Mechanical ventilation within 48 h, n (%)	36 (100)	51 (85)	0.024
Mechanical ventilation (days)	36/36 14 [8–18]	50/60 11 [7–21]	0.385
ICU mortality, n (%)	29 (81)	20 (33)	< 0.0001

Data represent number of patients and percentage (%) or median [IQR]. For each parameter, if missing values are present, the number of available data is expressed. p values were calculated by non-parametric One-way ANOVA, χ^2^ test, or Fisher’s exact test, as appropriate

*BMI* body mass index, *SOFA* sequential organ failure assessment, *WBC* white blood cell count, *Mean PEEP* positive end expiratory pressure, for each patient, the average of the first 7 days after ICU admission was calculated, *C*_*dyn*_ dynamic respiratory system compliance, *PaO*_*2*_ arterial oxygen partial pressure, *FiO*_*2*_ fraction of inspired oxygen, *BUN* blood urea nitrogen, *CRP* C-Reactive protein, *ICU LOS* intensive care unit length of stay

### Inflammatory markers and AKI

**Fig. 2:**
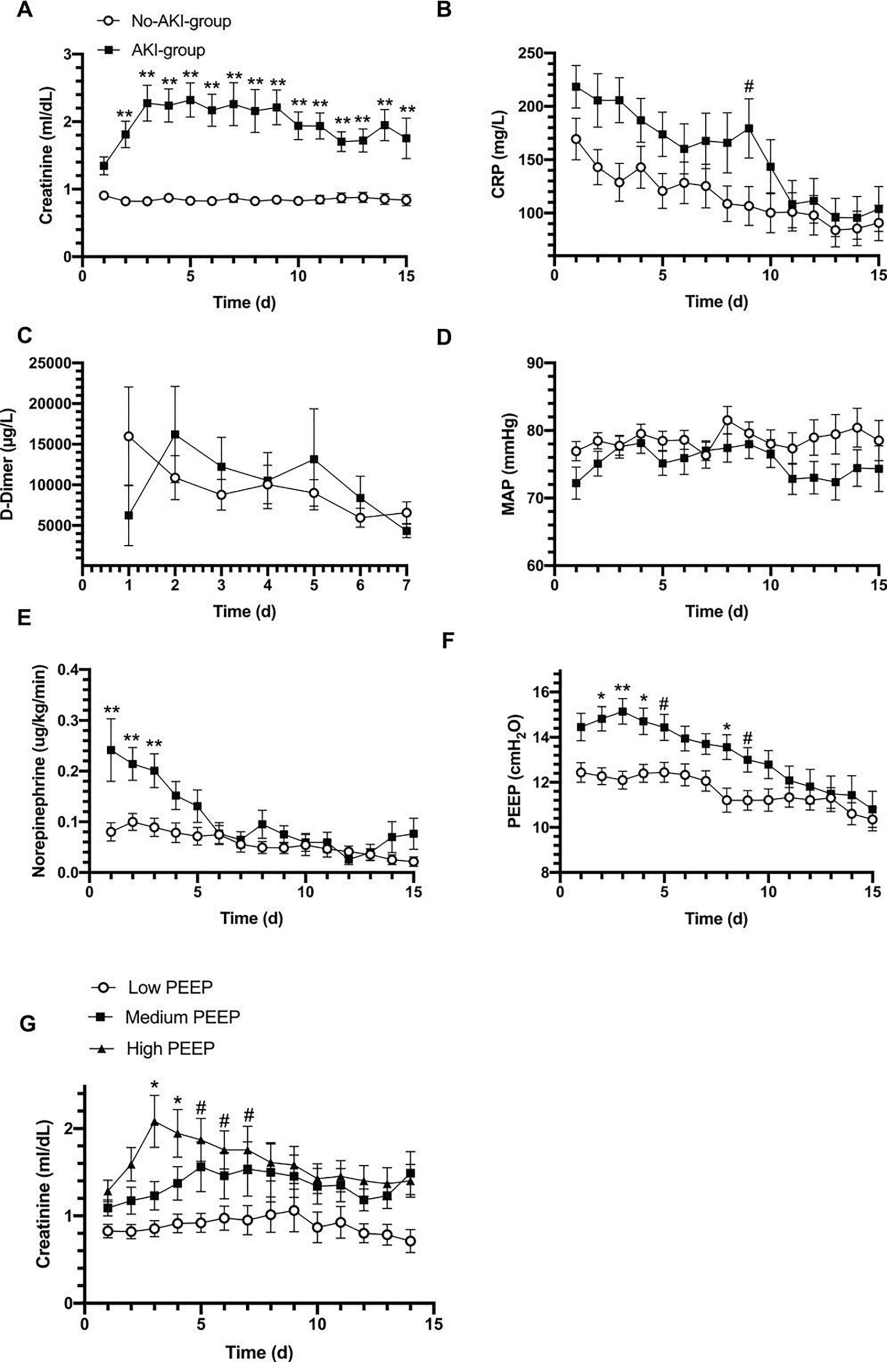
Laboratory data, PEEP and hemodynamics in patients who did or did not develop AKI. Trend in **A** creatinine, **B** C-reactive protein, **C** D-Dimer, **D** mean arterial pressure (MAP), **E** norepinephrine dose, **F** PEEP; **G** creatinine levels over time in patients treated with low (n = 31), medium (n = 32) or high (n = 32) PEEP. Two-way ANOVA. **p < 0.001, *p < 0.01, #p < 0.05. All data represent mean ± SEM

To investigate the possible relationship between the development of AKI and inflammation, we analyzed inflammatory markers and coagulation function in patients who developed AKI and those who did not. D-dimer levels on admission were similar between the two groups. However, upon admission, CRP was higher in patients who developed AKI compared to those who did not (238 [129–294] vs. 141 [68–255] mg/L, p = 0.027, Table 2), and tended to remain higher during the first 9 days after ICU admission (Fig. 2B).

### Hemodynamics and AKI

We examined the mean arterial pressure, vasopressor requirement, diuretic use, and fluid balance in patients divided by AKI group to evaluate the relationship between hemodynamics, fluid management, and renal function. During the first 15 days of ICU stay, there was no difference in mean arterial pressure in patients who developed AKI compared to those who did not (Fig. 2D). However, patients in the AKI group were treated with significantly higher doses of norepinephrine (Fig. 2E). Nevertheless, diuretic use (94% vs. 92%) and duration (7 [3–13] vs. 8 [3–13] days), cumulative urine output, and the total cumulative fluid balance over the 28 days of observation were similar in the AKI and no-AKI-groups (Table 2).

### Respiratory data and ventilatory management

Upon admission, the PaO_2_:FiO_2_ was similar in patients who developed AKI and patients who did not. The dynamic compliance of the respiratory system was similar in the two groups (31.4 [26.4–37.9] vs. 31.3 [26.4–35.3] mL/cmH_2_O, p = 0.754, Table 2). On admission and during the first week of ICU stay, patients who developed AKI were ventilated with higher PEEP (Fig. 2F). More frequently patients in the AKI-group underwent prone positioning (75% vs. 55%, p = 0.05) and for a longer duration (3 [1–5] vs. 1 [0–3] days, p = 0.015). Neuromuscular blockade was used for a longer duration in the AKI-group than in the no-AKI-group (9 [6–12] vs. 6 [1–11] days, p = 0.036), while no difference in the use of inhaled nitric oxide was found in the two groups (Table 2).

### Effect of PEEP level on kidney function and outcome

To further investigate any possible relationship between PEEP and the risk of developing AKI, we divided the entire population into tertiles of the average PEEP level applied during the first 7 days after admission. Patients in the low-PEEP group were treated with an average PEEP of 9.6 [8.0–10.5] cmH_2_O (n = 31), in the medium-PEEP group with PEEP 12.0 [11.2–12.7] cmH_2_O (n = 32), and in the high-PEEP group with PEEP 14.7 [13.7–16.7] cmH_2_O (n = 32) (Table 3). The PaO_2_:FiO_2_ on admission was similar in the three groups. However, the FiO_2_ was significantly higher in patients treated with high PEEP. Patients treated with high PEEP had higher SOFA scores on admission compared with patients with low PEEP (11 [8–12] vs. 8 [5–11], p = 0.057, Table 3).

**Table 3:** Clinical features and outcomes of the study population divided into tertiles of PEEP level applied during the first 7 days of ICU stay

	Low PEEP	Medium PEEP	High PEEP	p
	N = 31	N = 32	N = 32	
Baseline features				
Age (years)	60 [53–68]	61 [51–69]	61 [53–68]	0.478
Male sex (n-%)	20 (65)	26 (81)	29 (91)	0.037
SOFA score	31/31 8 [5–11]	32/32 9 [7–11]	32/32 11 [8–12]	0.057
Laboratory data				
PaO_2_:FiO_2_	31/31 124 [96–156]	32/32 111[92–148]	32/32 109 [79–142]	0.495
FiO_2_ (%)	31/31 0.7 [0.6–0.8]	32/32 0.8 [0.6–0.8]	32/32 0.8 [0.7–1.0]	0.028
Creatinine (mg/dL)	29/31 0.8 [0.6–0.9]	27/32 1.0 [0.7–1.3]	32/32 1.0 [0.9–1.5]	0.012
CRP (mg/L)	23/31 90 [45–172]	24/32 237 [143–325]	26/32 215 [109–282]	0.007
WBC	26/31 10,895 [5100–14040]	28/32 8420 [5970–10935]	28/32 8280 [6515–11345]	0.641
Ventilatory and diuretic data				
PEEP (cmH_2_O)	31/31 9.6 [8.0–10.5]	32/32 12.0 [11.2–12.7]	32/32 14.7 [13.7–16.7]	< 0.0001
Prone positioning, n (%)	5 (16)	8 (25)	10 (31)	0.372
C_dyn_ (mL/cmH_2_O)	23/31 26 [22–34]	26/32 31 [27–35]	29/32 32 [29–39]	0.057
Diuretic use, n (%)	29 (94)	30 (94)	29 (91)	0.999
Diuretic therapy duration (days)	31/31 6 [3–11]	32/32 11 [5–13]	32/32 7 [3–14]	0.486
Outcomes				
AKI-Risk, n (%)	3 (10)	7 (22)	4 (13)	
AKI-Injury, n (%)	1 (3)	4 (13)	3 (9)	
AKI-Failure, n (%)	4 (13)	8 (25)	16 (50)	
AKI-Injury + Failure, n (%)	5 (16)	12 (38)	19 (59)	0.002
CRRT, n (%)	1 (3)	6 (19)	12 (38)	0.003
ICU mortality, n (%)	13 (42)	14 (44)	22 (69)	0.057
Hospital mortality, n (%)	14 (48)	15 (52)	22 (71)	0.157

Data represent number of patients and percentage (%) or median [IQR]. p values were calculated by non-parametric ONE-way ANOVA, χ^2^ test, or Fisher’s exact test, as appropriate. Low-PEEP group: 9.6 [8.0–10.5] cmH_2_O; medium-PEEP group: 12.0 [11.2–12.7] cmH_2_O; high-PEEP group 14.7 [13.7–16.7] cmH_2_O

*PEEP* positive end expiratory pressure, *SOFA* sequential organ failure assessment, *PaO*_*2*_ arterial oxygen partial pressure, *FiO*_*2*_ fraction of inspired oxygen, *AKI* acute kidney injury, *CRRT* continuous renal replacement therapy

Patients treated with high PEEP had higher creatinine at baseline (Table 3) and creatinine further increased over time, whereas it remained significantly lower in patients treated with low PEEP (Fig. 2G). The incidence of AKI (injury + failure) in low-, medium-, and high-PEEP groups were 16%, 38%, and 59%, respectively (p = 0.002), while the need for CRRT occurred in 3%, 19%, and 38%, respectively (p = 0.003) (Table 3).

To assess whether PEEP could be an independent factor affecting AKI incidence, we performed a multivariate analysis in patients intubated within 48 h from ICU admission. Without any adjustment, the odds ratio of developing AKI in patients with high PEEP was significantly higher than in patients with low PEEP (OR = 8.3 [2.5–28.3] 95% CI p = 0.0007). After adjusting for confounders associated with both the development of AKI (Table 2) and the level of PEEP applied (Table 3) (including sex, SOFA score, serum creatinine, CRP at the time of ICU entry, and the presence of cardiovascular disease), the risk of developing AKI remained significantly higher in patients with high PEEP compared with those with low PEEP (OR = 4.96 [1.1–21.9] 95% CI p = 0.034) (Table 4).

**Table 4:** Multivariate analysis for risk of AKI in patients treated with low or high PEEP

Crude model					
Outcome	Comparisons	OR	95% CI		p value
Acute kidney	Medium vs low	3.75	0.98	14.39	0.0541
injury or failure	High vs. low	8.33	2.45	28.29	0.0007

Adjusted model					
Outcome	Comparisons	OR	95% CI		p value
Acute kidney injury or failure	Medium vs. low	1.91	0.35	10.48	0.454
	High vs low	4.96	1.13	21.91	0.034
	Confounders				
	Male	0.64	0.13	3.12	0.580
	SOFA score	1.09	0.91	1.32	0.347
	Creatinine	2.61	0.61	11.23	0.198
	CRP	1.00	1.00	1.01	0.744
	Cardiovascular disease	2.80	0.88	8.89	0.081

Logistic regression models to assess the association between AKI within 7 days after admission and average PEEP level applied during the first 7 days after admission modeling the probability of having AKI-Injury or failure against the likelihood of AKI-Risk and no-AKI. Multivariate analyses, adjusting for baseline characteristics known a priori to be potential confounders and factors associated with both exposure to PEEP and outcome. Odds ratio (OR), 95% CIs, and p values. Low-PEEP group: 9.6 [8.0–10.5] cmH_2_O; medium-PEEP group: 12.0 [11.2–12.7] cmH_2_O; high-PEEP group 14.7 [13.7–16.7] cmH_2_O

The ICU mortality in patients with high PEEP was higher than in patients with low or medium PEEP, although not statistically significant (69% vs. 44% and 42% respectively, p = 0.057) (Table 3).

## Discussion

This study describes the demographic and clinical characteristics of 101 ICU patients with Covid-19 hypoxemic respiratory failure, mainly focusing on the clinical course, laboratory findings, and ventilatory management related to AKI development. The mortality rate in our patient population was 51.5%, with a greater risk of death in male patients and patients with pre-existing cardiovascular disease or cancer. Many patients received prone positioning and neuromuscular blockade, with a prolonged ICU course and extended duration of mechanical ventilation. Patients treated with high PEEP levels had a higher incidence of AKI and requirement of CRRT (Supplementary Fig. 1).

The 51.5% mortality rate observed in our study is consistent with previous reports [7, 14–16]. Whether patients with Covid-19 lung disease present with a clinical picture resembling typical ARDS or rather have a distinct disease has been the subject of debate [17, 18]. Despite the presence of hypoxemia and bilateral infiltrates on chest X-rays, the respiratory system compliance in the early phases of the disease is often preserved, with CT scan images frequently showing maintained lung aeration and low weight [19].

Among hospitalized Covid-19 patients, AKI is a severe and common complication. In this study, we observed a 38% incidence of AKI (KDIGO stage 2 and 3) [7, 8]. AKI was associated with a 2.5-fold increase in ICU mortality. A meta-analysis of 39 studies (25,566 patients) including ICU and non-ICU patients showed a pooled incidence of AKI of 15.4% with significant heterogeneity. However, the AKI incidence in patients with severe Covid-19 was 53% [8]. Prior studies in patients with all-cause ARDS showed an incidence of AKI in the 30–45% range [20, 21], while 17.7% of patients with H1N1 ARDS developed AKI [22]. In a recent study on AKI risk factors in patients with ARDS, no association was found between ventilatory parameters and the presence of AKI. In contrast, ‘ARDS-related’ risk factors associated with AKI were sepsis, non-cardiogenic shock, transfusion-related acute lung injury (TRALI), and pancreatitis [12]. These pathologies were rare in our population, and other factors may have accounted for the high incidence of AKI.

Our histological observations do not support direct renal damage caused by the virus. A direct effect could be mediated by SARS-CoV-2 interaction with the ACE2 receptor, which is the cell-entry receptor for SARS-CoV-2, widely expressed in the renal epithelium [10]. SARS-CoV-2 nucleocapsid protein has been demonstrated in the kidneys of deceased patients with Covid-19 [23]. Werion et al. described specific manifestations of proximal tubule dysfunction, including low molecular weight proteinuria, aminoaciduria, and defective handling of uric acid and phosphate, associated with ultrastructural signs of tubular injury [24]. In post-mortem analysis of kidney tissue, Braun et al. [25] found SARS-CoV-2 RNA in 60% of 63 patients (72% of those with AKI), suggesting that viral renal tropism is associated with disease severity and AKI development. However, other studies failed to demonstrate the presence of the virus at the ultrastructural level in the kidneys in most patients. Schurink et al. [26] showed that sporadic SARS-CoV-2-positive cells were present in the tubular epithelium of the kidney in only 9% of patients at an early stage of the disease (8 days).

Other mechanisms may include kidney damage secondary to cytokine storm [9]. In addition, the strong inflammatory response mounted by some patients in response to the SARS-CoV-2 infection and the activation of the coagulation cascade and platelets may contribute to renal injury via inflammatory cells invasion and micro-thrombi formation. In our cohort, CRP was significantly higher in patients who developed AKI. They were treated with higher doses of vasopressors, which may have been related to the higher degree of inflammation and the hemodynamic effects of positive pressure ventilation (PPV) with higher PEEP levels.

The use of diuretics and PPV to correct hypoxemia may cause hypovolemia, venous stasis, and renal hypoperfusion, contributing to AKI development via hemodynamic mechanisms. In our study, diuretics were used in over 90% of the patients; however, no difference in diuretic use, duration, or fluid balance was found in patients who developed AKI compared with those who did not.

It has been previously demonstrated that PPV can affect renal function by reducing renal perfusion and glomerular filtration [27]. During PPV, the pulmonary resistance rises during inspiration, causing an impaired emptying of the right ventricle. Also, the increased intrathoracic pressure and the displacement of the diaphragm towards the abdominal organs and vessels causes a reduction of venous return. In turn, these changes produce a decrease in cardiac output [28].

Previous studies on ARDS patients (who typically had low respiratory system compliance) did not find any relationship between PEEP levels and AKI [12]. Thus, in patients with Covid-19 presenting with lung compliance higher than the typical ARDS patient, the effect of PEEP on renal perfusion and glomerular filtration may be more pronounced, and the use of high PEEP may contribute to the development of renal dysfunction. This hypothesis is supported by the finding of a more pronounced impairment in renal blood flow in mechanically ventilated patients with SARS-CoV-2 ARDS, compared with patients with “classical” ARDS [29].

Our study found that patients who developed AKI were treated with higher PEEP levels than those who did not. Also, they more frequently underwent prone positioning and paralysis, had higher inflammatory markers, and were treated with higher doses of vasopressors. All these factors were likely related to a higher degree of inflammation and a more severe disease presentation in patients who developed AKI.

To investigate whether PEEP could be an independent factor associated with the development of AKI, we divided the population by the average PEEP level applied during the first 7 days of ICU stay, and we found that patients treated with higher PEEP more frequently developed AKI compared to patients who were treated with lower PEEP levels. To account for differences in disease severity and baseline characteristics, we performed a multivariate analysis correcting for confounding factors associated with both the exposure to PEEP and outcome (AKI injury or failure). These included sexes, SOFA score, serum creatinine, CRP at the time of ICU entry, and the presence of cardiovascular disease. We found that in patients exposed to high levels of PEEP, the odds of developing AKI were five times greater than the odds for those exposed to low levels of PEEP; moreover, for those exposed to medium levels of PEEP, the odds of developing AKI were two times greater than the odds for those exposed to low levels of PEEP.

These results suggest that in this patient population, a specific PEEP level should be set with caution, as the beneficial effect of higher PEEP on oxygenation may be counteracted by the harmful effects of overdistension and hemodynamic impairment, with negative consequences on renal function and outcome.

This study has some limitations. First, it is a single-center retrospective study, and any observed effect of various treatments on outcomes should be acknowledged cautiously. Second, patients were included in this study at the beginning of the pandemic, when steroids, monoclonal antibodies, anti-viral, and other adjunctive therapies were not available, and there was no evidence of their benefit. Third, given the rapidly changing guidelines at the beginning of the pandemic, differences in treatment over time could reflect changes in clinical management not accounted for in the analysis. Fourth, histology findings are presented for only two representative patients, limiting the generalizability of the results. Finally, the association between the use of high PEEP and the risk of developing AKI is only descriptive in nature since comparisons were not powered for hypothesis testing.

In conclusion, our findings suggest that the use of high-PEEP ventilation in Covid-19 ICU patients with hypoxemic respiratory failure may be linked to a higher risk of developing AKI, which in turn further increases mortality in patients who are already at increased risk of an unfavorable outcome.

## Supplementary Information

Below is the link to the electronic supplementary material.

Supplementary file1 (PDF 54 kb)

Supplementary file2 (DOCX 30 kb)

Supplementary file3 (DOCX 27 kb)

## Data Availability

The database is available on request.
