# Supplementary material for: Acute kidney injury (AKI) in patients with Covid-19 infection is associated with ventilatory management with elevated positive end-expiratory pressure (PEEP)
Source: J Nephrol. 2021 Jun 25;35(1):99–111. doi: 10.1007/s40620-021-01100-3 (PMC8226340; doi:10.1007/s40620-021-01100-3)

Supplementary Figure 1 - PEEP level and incidence of CRRT during the study period

A) PEEP level applied during the first week of ICU stay in patients divided by week of admission (1 to 10, see text for details), *p<0.01 vs weeks 1 to 5, # p<0.05 vs weeks 2 to 4, One Way ANOVA. B) Incidence of CRRT by week of admission. In panel A the horizontal line represents median, the box plot represents interquartile range and the bars represent minimum and maximum values

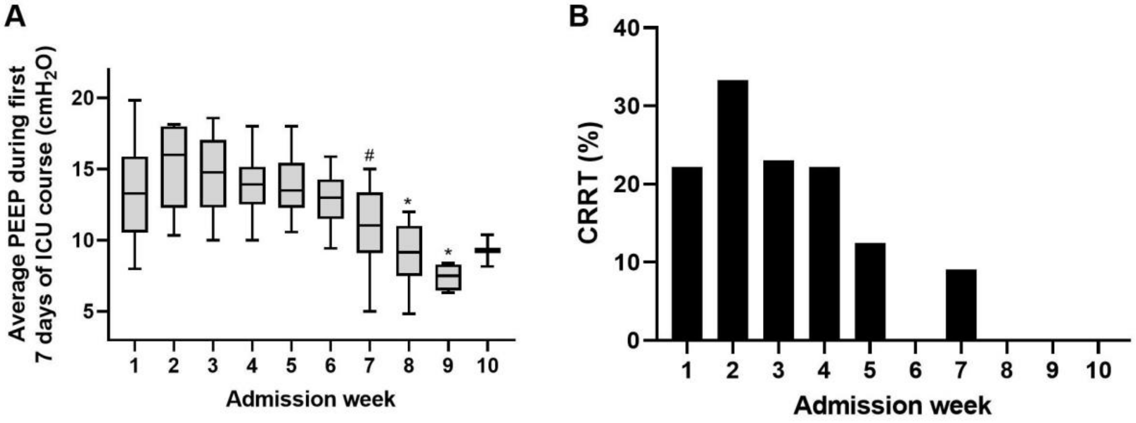

Supplement: Supplementary file 1 — Supplementary file1 (PDF 54 kb) [file 40620_2021_1100_MOESM1_ESM.pdf]
