# Supplementary material for: Acute kidney injury (AKI) in patients with Covid-19 infection is associated with ventilatory management with elevated positive end-expiratory pressure (PEEP)
Source: J Nephrol. 2021 Jun 25;35(1):99–111. doi: 10.1007/s40620-021-01100-3 (PMC8226340; doi:10.1007/s40620-021-01100-3)
Supplement: Supplementary file 2 — Supplementary file2 (DOCX 30 kb) [file 40620_2021_1100_MOESM2_ESM.docx]

**Supplementary methods**

**Data Collection**

The following patients’ features were recorded: age, sex, height, and weight; body mass index (BMI) and ideal body weight (IBW) were calculated.

Past medical history: cardiovascular disease, respiratory disease, smoking, diabetes mellitus, and neoplastic disease. Laboratory tests: complete blood count with differential, basic metabolic panel and liver function tests, serum albumin concentration, and inflammation markers such as C-reactive protein and LDH. Arterial blood gas analysis values such as pH, partial pressure of oxygen, partial pressure of carbon dioxide, lactate concentration, and hemoglobin oxygen saturation were also recorded. In addition, daily total fluid balance mean arterial pressure, type and dose of vasoactive agents, and the use of diuretics were recorded. The following ventilatory parameters were recorded: mode of ventilation, mean daily PEEP, FiO_2_, mean daily Peak airway pressure, tidal volume, and respiratory rate. Airway plateau pressure was recorded when available. In addition, we recorded if patients were treated with continuous neuromuscular blockade, prone positioning, or nitric oxide.

**Calculations**

Dynamic respiratory system compliance (C_dyn_) = Vt (ml) / (Peak airway pressure - PEEP (cmH_2_O))

Static respiratory system compliance (C_stat_) = Vt (ml) / (Plateau airway pressure - PEEP (cmH_2_O))

Acute kidney injury was defined according to KDIGO criteria based on serum creatinine (for baseline serum creatinine, the first value within 48 hours from ICU admission was considered). Glasgow coma scale (GCS) and SOFA score were calculated at ICU admission according to standard formulae. If the data for the calculation were missing, the values of the following day were used. Sedated and paralyzed patients were assigned a GCS of 3.
